# Factors associated with low back pain in patients with lumbar spinal stenosis: a cross-sectional study

**DOI:** 10.1186/s12891-022-05483-7

**Published:** 2022-06-08

**Authors:** Izaya Ogon, Atsushi Teramoto, Hiroyuki Takashima, Yoshinori Terashima, Mitsunori Yoshimoto, Makoto Emori, Kousuke Iba, Tsuneo Takebayashi, Toshihiko Yamashita

**Affiliations:** 1 Department of Orthopaedic Surgery, Sapporo Medical University School of Medicine, 291, South-1, West-16, Chuo-ku, Sapporo, 060-8543 Japan; 2 Department of Orthopaedic Surgery, Sapporo Maruyama Orthopaedic Hospital, 1-3, North-7, West-27, Chuo-ku, Sapporo, 060-0007 Japan

**Keywords:** Lumbar spinal stenosis, Low back pain, Spinopelvic alignment, Sagittal vertical axis, Pelvic incidence-lumbar lordosis, Magnetic resonance imaging, T2 mapping

## Abstract

**Background:**

Low back pain (LBP) is a major symptom of symptomatic lumbar spinal stenosis (SLSS). It is important to assess LBP in patients with SLSS to develop better treatment. This study aimed to analyse the factors associated with LBP in patients with SLSS.

**Methods:**

This cross-sectional study included consecutive patients with SLSS aged between 51 and 79 years who had symptoms in one or both the legs, with and without LBP. The participants were classified into two groups: the high group (LBP visual analogue scale [VAS] score ≥ 30 mm) and the low group (LBP VAS score < 30 mm). We performed multiple logistic regression analysis with the high and low groups as dependent variables, and a receiver operating characteristic (ROC) analysis.

**Results:**

A total of 80 patients with LSS were included (35 men and 45 women; mean age 64.5 years), with 47 and 30 patients in the high and low groups, respectively. Multivariate logistic regression analysis revealed that the sagittal vertical axis (SVA; + 10 mm; odds ratio, 1.331; 95% confidence interval, 1.051 − 1.660) and pelvic incidence-lumbar lordosis (PI-LL; + 1°; odds ratio, 1.065; 95% confidence interval, 1.019–1.168) were significantly associated with LBP. A receiver operating characteristic analysis revealed cut-off values of 47.0 mm of SVA and 30.5° of PI-LL, respectively.

**Conclusion:**

Our results indicated that SVA and PI-LL were significant predictors for LBP in SLSS. It is suggested that these parameters should be taken into consideration when assessing LBP in patients with SLSS.

## Background

Symptomatic lumbar spinal stenosis (SLSS) is characterized by symptoms such as low back pain (LBP), pain and numbness in the lower extremities, and neurogenic claudication [1]. Amundsen et al. [2, 3] reported that the most common symptoms in patients with SLSS were back pain, including LBP (prevalence, 95%); claudication (91%); leg pain (71%); weakness (33%); and voiding disturbances (12%). Miyakoshi et al. [4] reported that the prevalence of SLSS among individuals aged ≥ 50 years in a rural Japanese cohort was 10.8% and that the prevalence of SLSS with chronic low back pain (CLBP) and SLSS without CLBP were 67.6% and 32.4%, respectively. Therefore, LBP is considered a major symptom of SLSS.

Lumbar spinal stenosis (LSS) is a degenerative condition that involves spinal canal narrowing due to facet joint osteoarthritis, ligamentum flavum hypertrophy, intervertebral disc bulging, and spondylolisthesis [1]. These spondylotic changes can induce LBP, which can negatively affect the patients’ quality of life [5, 6]. Kobayashi et al. [6] used the Japanese Orthopaedic Association Back Pain Evaluation Questionnaire to compare the quality of life of patients with LBP, with and without SLSS. They showed that the scores for pain-related disorder, gait disturbance, social life disturbance, and psychological disorders were significantly lower in patients with SLSS as compared to those without SLSS. Therefore, to develop better treatments in the future, it is important to assess LBP in patients with SLSS.

Recent studies have investigated the mechanism of LBP and found that the following factors contribute to LBP: overweight [7], osteoporosis [8], spondylolisthesis [9], range of motion [10], spinopelvic alignment [11], muscle degeneration [12–17], intervertebral disc degeneration [18, 19], Modic changes [20], and facet joint degeneration [21]. Based on these findings, this study aimed to analyse the factors associated with LBP in patients with SLSS. The following factors were evaluated: lower extremity symptoms, body mass index, bone mineral density, spondylolisthesis, range of motion, spinopelvic alignment, cross-sectional area and fat infiltration of the multifidus muscle, intervertebral disc degeneration, Modic changes, and facet joint degeneration. Additionally, ROC analysis was performed to calculate cutoff values for variables that were found to be associated.

## Methods

The study was performed in accordance with the Declaration of Helsinki after approval by the Ethics Committee of our institution (approval number: 262–1074). All participants received written and verbal explanations of the study and provided informed consent before participation.

### Participants

The diagnostic criteria for symptomatic SLSS were based on the SLSS definition from the North American Spine Society guidelines [22]. The patients who visited our university hospital from 2015 to 2016 were interviewed by well-experienced spine surgeons (I.O., Y.T., M.Y., and T.T.). The surgeons performed physical testing on consecutive patients who reported with symptoms that were induced or exacerbated with walking or prolonged standing and relieved with lumbar flexion, sitting, and recumbency pain, numbness, and neurological deficits in the lower extremities and buttocks as well as bladder or bowel dysfunction. Thereafter, radiographic and MRI findings suggestive of degenerative stenosis of the spinal canal or intervertebral foramen were correlated with the reported symptoms and clinical findings that confirmed the diagnosis of LSS. The same surgeons made the final diagnosis of symptomatic SLSS, which necessitated the presentation of both clinical symptoms and radiographic findings of LSS. All patients with a confirmed diagnosis of SLSS were recruited as participants. The exclusion criteria were as follows: aged < 50 and > 80 years old, acute trauma, infection, neoplasm, history of spinal surgery or spinal fracture, LBP with symptom presentation for < 3 months, and spondylolisthesis with obvious instability, which was defined as a sagittal translation of ≥ 3 mm, segmental mobility of ≥ 20°, or posterior opening of ≥ 5° on flexion/extension radiographs. CLBP was defined as pain, discomfort, and stiffness in the lower back extending from the 12th rib to the lumbar or lumbosacral area for more than three months. All participants rated their LBP on the visual analogue scale (VAS; 0 − 100 mm).

### Outcomes

Based on the findings of previous reports [23–25], a VAS score of > 30 mm was classified as moderate or severe pain (the high group), whereas that of ≤ 30 mm was classified as no or mild pain (the low group).

### Classification of lower extremity symptoms

As described in a previous report [26], lower extremity symptoms were classified as cauda equina, radicular pain, and mixed by the surgeons (I.O., Y.T., M.Y., and T.T.) based on history, physical examination, and MRI findings. The radicular type was characterized by symptoms of pain, burning, numbness, and paresthesia following a specific dermatome with radiological evidence of the responsible nerve root compression, which was confirmed if intermittent claudication was abolished following single nerve root infiltration. Patients of the cauda equina type presented bilateral symptoms related to cauda equina compression syndrome with less dermatomal-specific neurogenic claudication and radiological evidence of cauda equina compression.

### Measurement of body mass index and bone mineral density

We measured the patients’ height and body weight; this was used to calculate their body mass index (kg/m^2^) [27]. Bone mineral density was measured at L2, L3, and L4 using a dual-energy X-ray absorptiometry scanner simultaneously with radiographs and MRI.

### Measurement of range of motion

We acquired dynamic flexion–extension radiographs of the participants in the standing position. The angle between the superior endplates of L1 and S1 was termed the range of motion.

### Measurement of the sagittal spinopelvic radiologic parameters

**Fig. 1:**
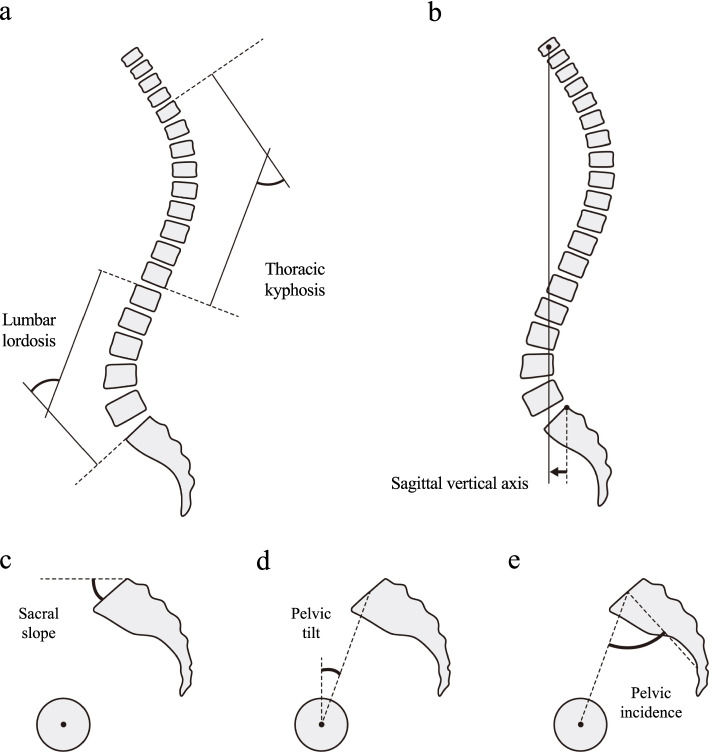
Measurement of the sagittal spinopelvic parameters. **a** Lumbar lordosis is measured from the superior endplate of L1 to the superior endplate of S1, and thoracic kyphosis is measured from the superior endplate of T4 to the inferior endplate of T12. **b** Sagittal vertical axis is the horizontal offset from the posterosuperior edge of S1 to the body of C7. **c** Sacral slope is the angle between the horizontal and the superior sacral endplate. **d** Pelvic tilt is the angle between the vertical axis and the line running from the midpoint of the sacral plate to the centre of the femoral head axis. (**e**) Pelvic incidence is the angle between a line perpendicular to the superior sacral endplate at its midpoint and the line connecting this point to the centre of the femoral head axis

As described in a previous report, we obtained full-length spinal and pelvic radiographs of the participants in the standing position and used them to calculate several parameters [11]. The following sagittal spinal parameters were measured on sagittal-view spinal radiographs: lumbar lordosis (LL; the superior endplate of L1 to the superior endplate of S1; Fig. 1a), thoracic kyphosis (the superior endplate of T4 to the inferior endplate of T12; Fig. 1a), and sagittal vertical axis (SVA; the horizontal offset from the posterosuperior edge of S1 to the centre of the body of C7; Fig. 1b). The following sagittal pelvic parameters were measured on sagittal-view pelvic radiographs: sacral slope (angle between the horizontal and the superior sacral endplate; Fig. 1c), pelvic tilt (the angle between the vertical axis and the line running from the midpoint of the sacral plate to the centre of the femoral head axis; Fig. 1d), pelvic incidence (PI; angle between a line perpendicular to the superior sacral endplate at its midpoint and the line connecting this point to the centre of the femoral head axis; Fig. 1e), and PI-LL. Two investigators blinded to the study assessed the intraobserver and interobserver reliability of the measurements of the spinopelvic parameters (observer 1, I.O., and observer 2, H.T.). The κ values for intraobserver and interobserver reliability were as follows: LL, 0.85 and 0.91; thoracic kyphosis, 0.89 and 0.92; SVA, 0.84 and 0.91; SS, 0.83 and 0.90; pelvic tilt, 0.85 and 0.88; PI, 0.81 and 0.87; and PI-LL, 0.80 and 0.84, respectively.

### Measurement of the cross-sectional area and fat infiltration of the multifidus muscle

We used the Signa HDx 1.5 T magnetic resonance imaging (MRI) system (GE Healthcare, Milwaukee, WI, USA) with a spine coil to obtain T2-weighted MRI images. The cross-sectional area and fat infiltration of the multifidus muscle at the L3–L4, L4–L5, and L5–S levels were measured using axial T2-weighted MRI. The cross-sectional area was assessed by manually tracing the fascial border of the multifidus muscle, as previously described [14]. We analysed the histograms of signal intensity in the regions of interest for the areas using digitized image-processing software (Image J; National Institutes of Health, Bethesda, MD, USA). We measured the percentage area with fat infiltration using the software’s pseudo-colouring tool, which causes pixels representing fat tissue to appear red. We then calculated the percentage of the muscle compartment that was red. The cross-sectional area and fat infiltration data were averaged between the right and left multifidus muscles. The κ values for intraobserver and interobserver reliability were 0.88 and 0.92 for cross-sectional area and 0.82 and 0.89 for fat infiltration, respectively.

### Assessment of intervertebral disc degeneration using T2 mapping

We performed MRI T2 mapping using a protocol described in previous studies [28–32]. Sagittal images were acquired with the patients in the supine position, and T2 maps were created on a pixel-by-pixel basis. We used the T2 values of the midsagittal section, which was centred on the lumbar midline, with an optimized 8-echo multi-spin echo sequence obtained using the Advantage Workstation (version 4.4, Functool; GE Healthcare, Milwaukee, WI, USA) with the following parameters: repetition time, 1000 ms; first echo time, 14.8 ms; last echo time, 118.6 ms; receiver bandwidth, ± 15.63 kHz; field of view, 22 cm; matrix, 320 × 256; slice thickness/gap, 4 mm/4 mm; number of slices, 5; number of excitations, 2; and total scan time, 8 min and 34 s. We did not use the first echo from the multi-spin to minimize the effect of the stimulated echo. The T2 map was calculated for each pixel from the signal intensity in the respective echo time using the following formula: signal intensity (echo time) = e ^– echo time /T2^.

**Fig. 2:**
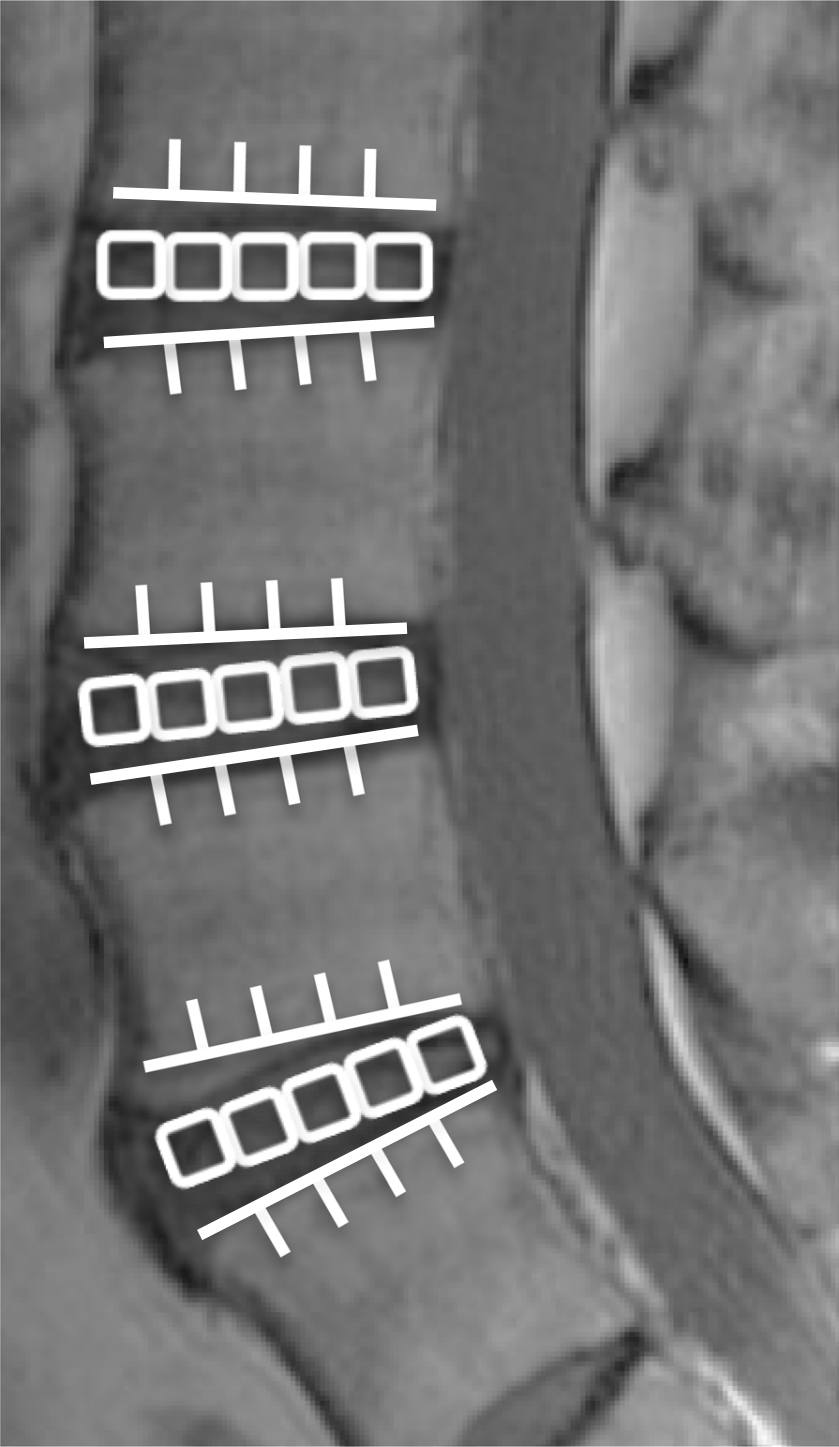
Intervertebral discs at L3–L4, L4–L5, and L5–S1 were divided into five equal areas each, with the first, middle, and last fifth areas being the anterior annulus fibrosus, the centre of the nucleus pulposus, and the posterior annulus fibrosus, respectively

The intervertebral discs at L3–L4, L4–L5, and L5-S were divided into five equal areas each. We measured the mean T2 values at the first, middle, and last fifth areas, which were the anterior annulus fibrosus, the centre of the nucleus pulposus, and the posterior annulus fibrosus, respectively [28–32] (Fig. 2) A total of 300 levels were evaluated. The T2 values were measured using MedCalc (version 10.2.0.0; MedCalc Software, Mariakerke, Belgium) by a PhD researcher (H.T.) with 15 years of experience in spinal MRI analysis.

### Assessment of Modic changes

Modic changes were evaluated from L1–L2 to L5–S1 and classified as none, type 1, type 2, or type 3, according to their signal patterns on T1- and T2-weighted sagittal MRI [33]. Type 1 Modic changes were hypointense on T1-weighted images and hyperintense on T2-weighted images. Type 2 Modic changes were hyperintense on both T1- and T2-weighted images. Type 3 Modic changes were hypointense on both T1- and T2-weighted images. The intraobserver and interobserver reliability were excellent, with κ values of 0.81 and 0.84, respectively.

### Assessment of facet joint degeneration

To evaluate facet joint degeneration, we acquired axial images at three lumbar levels (L3–L4, L4–L5, and L5−S1) using computed tomography (Aquilion PRIME, Toshiba, Japan). As described in a previous report, facet joint degeneration was classified into four grades: grade 0, normal; grade 1, mild degenerative disease; grade 2, moderate degenerative disease; and grade 3, severe degenerative disease [34]. If there was a difference in facet joint degeneration severity between the right and left sides at the same lumbar level, the worse grade was recorded. In all patients, this severity was categorized as either grade 0–1 or grade 2–3. The intraobserver and interobserver reliability were excellent, with κ values of 0.80 and 0.81, respectively.

### Statistical analyses

We compared the body mass index, lower extremity symptoms, VAS scores of lower extremity pain and numbness, bone mineral density, spondylolisthesis, range of motion, spinopelvic alignment, cross-sectional area and fat infiltration of the multifidus muscle, intervertebral disc degeneration, Modic changes, and facet joint degeneration between the high and low groups using the Mann − Whitney U test and the chi-square test. The high and low groups were defined as the dependent variables (high = 1, low = 0), and the crude odds ratios (OR) were calculated by using univariate logistic regression analysis without adjustment. Multivariate logistic regression was used to calculate adjusted OR with 95% confidence interval (CI) after controlling simultaneously for potential confounders (*p* < 0.10 in the univariate logistic regression analysis). As listed in Table 1, age, body mass index, VAS scores of lower extremity pain and numbness, bone mineral density, range of motion, thoracic kyphosis, LL, SVA, sacral slope, pelvic tilt, PI, PI-LL, cross sectional area, fat infiltration, and T2 value were included in the independent variables as continuous scale, and ORs were calculated for each 1-unit increase. Sex, lower extremity symptoms, spondylolisthesis, Modic change, and facet degeneration were included as independent variables as nominal scale, and ORs were calculated. We also performed a receiver operating characteristic (ROC) analysis of the significant variables to determine the boundary values of the VAS score for LBP. We used the Youden index to determine the optimal cut-off point for a test [35]. The Youden index is defined as the maximum vertical distance between the ROC curve and the diagonal or chance line and is calculated as Youden index = maximum {sensitivity + specificity − 1}. Using this measure, the cut-off point on the ROC curve which corresponds to the Youden index, that is, at which (sensitivity + specificity − 1) is maximized, is taken to be the optimal cut-off point. Statistical significance was set at *p* < 0.05. We used SPSS (version 27.0; IBM Corp., Armonk, NY, USA) for all statistical analyses. Numerical data are expressed as mean ± standard error of the mean. If the number of variables ultimately incorporated into the model for multivariate logistic regression analysis would be 3, we expected a 4:3 ratio of people to be classified as the low and high groups. Based on this, the required sample size was calculated to be 70 patients.

**Table 1:** The reference standards of continuous and nominal scales as independent variables in multiple logistic regression analyses

**Continuous scale**	**One-unit increase**
Age (years)	Per 1 year
Body mass index (kg/m2)	Per 1 kg/m^2^
Visual analogue scale (mm)	
Low back pain	Per 1 mm
Lower extremity pain	Per 1 mm
Lower extremity numbness	Per 1 mm
Bone mineral density (g/cm2)	Per 0.1 g/cm^2^
Range of motion (°)	Per 1°
Spinopelvic alignment	
Thoracic kyphosis (°)	Per 1°
Lumbar lordosis (°)	Per 1°
Sagittal vertical axis (mm)	Per 10 mm
Sacral slope (°)	Per 1°
Pelvic tilt (°)	Per 1°
Pelvic incidence (°)	Per 1°
Pelvic incidence − Lumbar lordosis (°)	Per 1°
Cross sectional area (mm2)	
L3 − L4	Per 10mm^2^
L4 − L5	Per 10mm^2^
L5 − S1	Per 10mm^2^
Fat infiltration (%)	
L3 − L4	Per 1%
L4 − L5	Per 1%
L5 − S1	Per 1%
T2 value (ms)	
L3 − L4	
Anterior annulus fibrosus	Per 1 ms
Nucleus pulposus	Per 1 ms
Posterior annulus fibrosus	Per 1 ms
L4 − L5	
Anterior annulus fibrosus	Per 1 ms
Nucleus pulposus	Per 1 ms
Posterior annulus fibrosus	Per 1 ms
L5 − S1	
Anterior annulus fibrosus	Per 1 ms
Nucleus pulposus	Per 1 ms
Posterior annulus fibrosus	Per 1 ms
**Nominal scale**	
Sex	
Male	
Female (reference)	
Lower extremity symptoms	
Cauda equina	
Radicular pain	
Mixed	
Spondylolisthesis	
Yes	
No (reference)	
Modic change	
None	
Type 1	
Type 2	
Type 3	
Facet joint degeneration	
L3 − 4	
Grade0 − 1	
Grade2 − 3	
L4 − 5	
Grade0 − 1	
Grade2 − 3	
L5 − S	
Grade0 − 1	
Grade2 − 3	

## Results

We found that 80 patients (mean age 64.5 ± 1.8 years; range, 51–79 years) satisfied the inclusion criteria. As shown in Table 2, the high group included 47 patients (67.1%; 21 males and 26 females), and the low group included 33 patients (32.9%; 11 males and 12 females). The mean ages and body mass indices of the high and low groups were not statistically significant. The type of lower extremity for cauda equina, radicular pain, and mixed were 19, 18, and 10 cases in the high groups, and 15, 10, and 8 cases in the low group, respectively. There was no difference in the fraction of lower extremity symptoms between the high and low groups. The high group had significantly higher mean VAS scores for LBP, and lower extremity pain and numbness than in the low group (*p* < 0.01). Moreover, there were no significant between-group differences in bone mineral density, frequency of occurrence of spondylolisthesis, and range of motion. The spinopelvic parameters showed statistically significant differences with regards to LL, SVA, and PI-LL between the two groups (LL, *p* < 0.01; SVA, *p* < 0.01; PI-LL, *p* < 0.01). Also, there was a significant between-group difference in the T2 value of the posterior annulus fibrosus at the L4–L5 level (*p* < 0.01), regarding the T2 values of the discs. There were no significant between-group differences in cross-sectional area and fat infiltration, frequency of occurrence of Modic changes, and facet joint degeneration.

**Table 2:** Comparison of variables between the high and low groups

	High (n = 47)	Low (n = 33)	*p*
Age (years)	65.1 ± 1.6	63.7 ± 1.9	0.71*
Sex			
Male	21	14	0.84**
Female	26	19	
Body mass index (kg/m^2^)	23.2 ± 0.7	24.6 ± 0.9	0.38*
Lower extremity symptoms			0.76**
Cauda equina	19	15	
Radicular pain	18	10	
Mixed	10	8	
Visual analogue scale (mm)			
Low back pain	62.6 ± 2.0	16.1 ± 1.1	< 0.01*
Lower extremity pain	74.3 ± 4.8	50.1 ± 3.3	< 0.01*
Lower extremity numbness	75.1 ± 5.0	49.8 ± 4.0	< 0.01*
Bone mineral density (g/cm^2^)	1.03 ± 0.04	1.11 ± 0.09	0.40*
Spondylolisthesis			
Yes	11	7	0.82**
No	36	26	
Range of motion (°)	41.2 ± 3.3	43.5 ± 4.1	0.47*
Spinopelvic alignment			
Thoracic kyphosis (°)	26.1 ± 1.3	28.9 ± 2.2	0.46*
Lumbar lordosis (°)	31.5 ± 2.9	39.5 ± 3.3	< 0.01*
Sagittal vertical axis (mm)	56.1 ± 6.7	29.8 ± 6.1	< 0.01*
Sacral slope (°)	27.5 ± 1.4	31.1 ± 1.3	0.24*
Pelvic tilt (°)	19.7 ± 1.4	17.8 ± 1.1	0.39*
Pelvic incidence (°)	47.2 ± 1.6	48.9 ± 2.0	0.65*
Pelvic incidence − Lumbar lordosis (°)	15.7 ± 1.1	9.4 ± 0.9	< 0.01*
Cross sectional area (mm^2^)			
L3 − L4	392.6 ± 37.1	421.5 ± 39.9	0.71*
L4 − L5	421.5 ± 37.1	438.1 ± 40.4	0.50*
L5 − S1	443.8 ± 42.3	424.7 ± 40.1	0.59*
Fat infiltration (%)			
L3 − L4	12.6 ± 1.5	11.4 ± 1.2	0.78*
L4 − L5	19.1 ± 2.1	16.4 ± 1.7	0.41*
L5 − S1	19.9 ± 1.9	17.5 ± 1.8	0.52*
T2 value (ms)			
L3 − L4			
Anterior annulus fibrosus	60.9 ± 1.8	61.7 ± 1.3	0.72*
Nucleus pulposus	64.2 ± 3.0	62.6 ± 1.5	0.62*
Posterior annulus fibrosus	55.9 ± 1.9	56.8 ± 1.4	0.46*
L4 − L5			
Anterior annulus fibrosus	58.1 ± 2.1	59.1 ± 3.0	0.50*
Nucleus pulposus	59.7 ± 3.0	60.2 ± 2.4	0.39*
Posterior annulus fibrosus	51.3 ± 1.5	58.2 ± 2.1	< 0.01*
L5 − S1			
Anterior annulus fibrosus	59.5 ± 2.0	59.0 ± 2.3	0.50*
Nucleus pulposus	62.1 ± 2.7	59.6 ± 2.1	0.37*
Posterior annulus fibrosus	55.2 ± 1.9	56.6 ± 1.8	0.41*
Modic change			
None	33	23	0.70**
Type 1	3	1	
Type 2	10	9	
Type 3	1	0	
Facet joint degeneration			
L3 − L4			
Grade 0 − 1	31	19	0.45**
Grade 2 − 3	16	14	
L4 − L5			
Grade 0 − 1	17	13	0.77**
Grade 2 − 3	30	20	
L5 − S1			
Grade 0 − 1	22	15	0.90**
Grade 2 − 3	25	18	

Participants with an LBP VAS of > 30 mm and were ≤ 30 mm classified into the high and the low groups, respectively

All numerical data are presented as means ± standard error of the mean values

^*^ Mann–Whitney U test, ** chi-squared test

Table 3 shows the results of the multiple logistic regression analysis performed, with the high and low groups as dependent variables. SVA was significantly associated with LBP (+ 10 mm; OR, 1.318; 95% confidence interval, 1.041–1.650), and this association remained significant after adjusting for other significant variables (+ 10 mm; OR, 1.331; 95% CI, 1.051 − 1.660). PI-LL was significantly associated with LBP (+ 1°; OR, 1.064; 95% CI, 1.017–1.167), and this association remained significant after adjusting for other significant variables (+ 1°; OR, 1.065; 95% CI, 1.019–1.168).

**Table 3:** Results of the multiple logistic regression analyses

	Crude odds ratios	95% confidence interval	*p*	Adjusted odds ratio	95% confidence interval	*p*
Age (+ 1 year)	1.017	0.983–1.051	0.33			
Sex (male)	1.006	0.964–1.047	0.80			
Body mass index (+ 1 kg/m^2^)	1.007	0.895–1.136	0.93			
Lower extremity symptoms						
Cauda equina	0.905					
Radicular pain	0.961	0.676–1.867	0.20			
Mixed	1.000	0.701–2.133	0.15			
Visual analogue scale						
Lower extremity pain (+ 1 mm)	1.021	0.994–1.048	0.12			
Lower extremity numbness (+ 1 mm)	1.025	0.990–1.060	0.13			
Bone mineral density (+ 0.1 g/cm^2^)	0.944	0.803–1.082	0.39			
Spondylolisthesis (yes)	1.023	0.979–1.073	0.41			
Range of motion (+ 1°)	0.959	0.919–1.009	0.18			
Spinopelvic alignment						
Thoracic kyphosis (+ 1°)	0.994	0.968–1.023	0.29			
Lumbar lordosis (+ 1°)	0.970	0.955–1.005	0.09			
Sagittal vertical axis (+ 10 mm)	1.318	1.041–1.650	< 0.05	1.331	1.051–1.660	< 0.05
Sacral slope (+ 1°)	0.955	0.907–1.008	0.10			
Pelvic tilt (+ 1°)	1.039	0.984–1.106	0.16			
Pelvic incidence (+ 1°)	1.003	0.954–1.051	0.93			
Pelvic incidence -lumbar lordosis (+ 1°)	1.064	1.017–1.167	< 0.05	1.065	1.019–1.168	< 0.05
Cross sectional area						
L3 − 4 (+ 10 mm^2^)	0.997	0.914–1.065	0.94			
L4 − 5 (+ 10 mm^2^)	0.985	0.935–1.042	0.48			
L5 − S (+ 10 mm^2^)	0.982	0.924–1.046	0.56			
Fat infiltration						
L3 − 4 (+ 1%)	1.009	0.980–1.038	0.57			
L4 − 5 (+ 1%)	1.023	0.989–1.059	0.18			
L5 − S (+ 1%)	1.014	0.989–1.039	0.28			
T2 value						
L3 − 4 (+ 1 ms)						
Anterior annulus fibrosus	1.002	0.956–1.050	0.94			
Nucleus pulposus	1.008	0.984–1.032	0.55			
Posterior annulus fibrosus	0.989	0.932–1.049	0.45			
L4 − 5 (+ 1 ms)						
Anterior annulus fibrosus	1.006	0.990–1.022	0.45			
Nucleus pulposus	0.993	0.911–1.063	0.48			
Posterior annulus fibrosus	0.963	0.910–1.021	0.32			
L5 − S (+ 1 ms)						
Anterior annulus fibrosus	1.005	0.899–1.134	0.91			
Nucleus pulposus	0.985	0.943–1.029	0.49			
Posterior annulus fibrosus	0.986	0.937–1.037	0.58			
Modic change						
None	1.000					
Type 1	1.821	0.451–6.228	0.39			
Type 2	1.217	0.395–2.140	0.78			
Type 3	1.580	0.164–15.299	0.70			
Facet joint degeneration						
L3 − 4 (Grade2 − 3)	1.138	0.403–2.791	0.61			
L4 − 5 (Grade2 − 3)	1.241	0.315–5.925	0.69			
L5 − S (Grade2 − 3)	1.145	0.625–2.100	0.40			

Participants with an LBP VAS of > 30 mm and were ≤ 30 mm classified into the high and the low groups, respectively

The high and low group were the dependent variables

The crude odds ratios were calculated via univariate logistic regression analysis without adjustment

The adjusted odds ratios were calculated via multivariate logistic regression analysis using forward selection (likelihood ratio) after adjustment for variables with *p* < 0.10 in the univariate logistic regression analysis

**Fig. 3:**
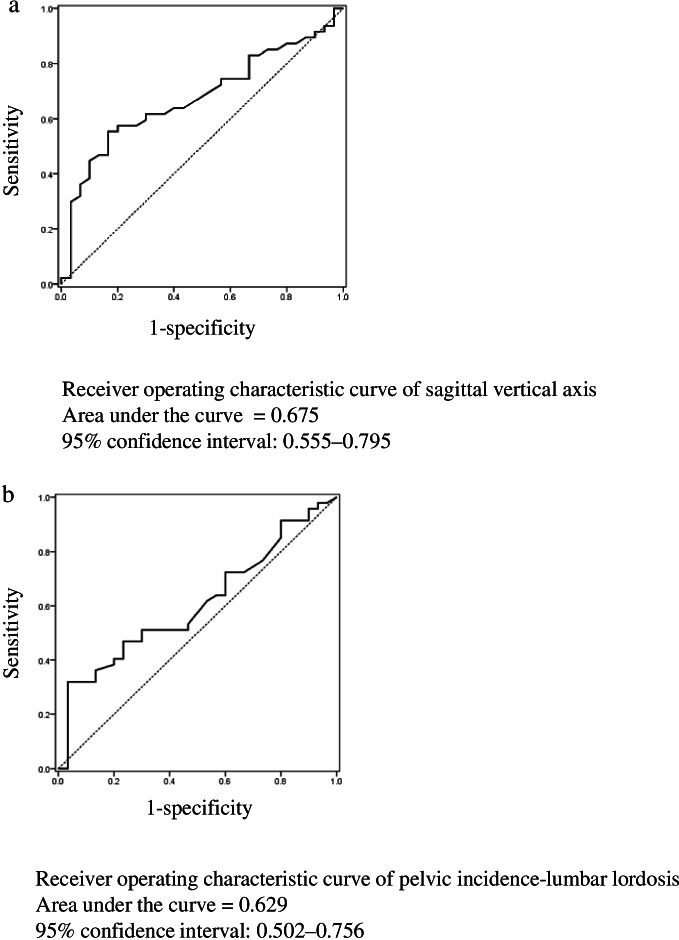
Receiver operating characteristic curves of sagittal vertical axis and pelvic incidence-lumbar lordosis. **a** The cut-off value for sagittal vertical axis was 47.0 mm. **b** The cut-off value for pelvic incidence-lumbar lordosis was 30.5°

The cut-off values obtained by using receiver operating characteristic analysis of SVA and PI-LL are shown in Fig. 3. The cut-off value, sensitivity, specificity, and area under the curve (AUC) for SVA were 47 mm, 55.3%, 83.3%, and 0.675, respectively (Fig. 3a). The cut-off value, sensitivity, specificity, and AUC for PI-LL were 30.5°, 31.9%, 96.7%, and 0.629, respectively (Fig. 3b).

## Discussion

This study investigated the factors associated with LBP in patients with SLSS, and found that SVA and PI-LL were significantly independently associated with LBP. SVA and PI-LL are important radiographic parameters found to be associated with LBP in spinal deformities [11]. Several reports have shown that SVA and/or PI-LL are associated with LBP in SLSS. Ogura et al. [36] classified patients with SLSS into two groups based on an SVA cut-off of 50 mm and compared their LBP numerical rating scale scores. The mean numerical rating scale scores in the group with an SVA ≥ 50 mm and in the group with an SVA < 50 mm were 6.6 and 5.6, respectively. Furthermore, the group with an SVA ≥ 50 mm had more severe LBP than did the group with an SVA < 50 mm, although this difference was not statistically significant (*p* = 0.058). Gao et al. [37] showed that patients with SLSS and degenerative scoliosis with a PI-LL > 20° had significantly higher Oswestry Disability Index scores, which indicated greater disability due to LBP, than those with a PI-LL < 20°. Miyakoshi et al. [4] reported that patients with SLSS with LBP were more likely to have a kyphotic lumbar spinal alignment and a stooped posture than those with SLSS without LBP. These findings are consistent with the result of this study, and SVA and PI-LL were important for assessing LBP in SLSS.

The present study cross-sectionally showed an association between LBP and SVA as well as between LBP and PI-LL, in SLSS, and indicated cut-off points of 47.0 mm for SVA and 30.5° for PI-LL. There have been reports of improved LBP and spinopelvic alignment after decompression surgery for SLSS [36, 38]. Ogura et al. [36] reported that, in patients with SLSS who underwent lumbar spinous process-splitting laminectomy, the mean SVA decreased from 42.5 mm preoperatively to 35.6 mm postoperatively, the mean PI-LL decreased from 14.9° preoperatively to 12.7° postoperatively, and the mean improvement in LBP numerical rating scale score was 3.3. Fujii et al. [38] showed that the mean symptom severity, assessed using the Zurich Claudication Questionnaire, decreased from 2.5 preoperatively to 1.7 postoperatively, mean SVA decreased from 49.1 mm preoperatively to 28.6 mm postoperatively, and mean PI-LL decreased from 12 mm preoperatively to 6 mm postoperatively in patients with SLSS who underwent recapping laminoplasty. In this study, AUC for the prediction was < 0.7, which is likely due to the low sensitivity. Analyses of ROC and AUC were performed to predict the reliability of the model. Values of ≥ 0.7 were considered clinically useful [39]. Therefore, it should be noted that because of the low sensitivity of the results in this study, a certain percentage of cases will fail to detect even below the cutoff values of 47.0 mm of SVA and 30.5° of PI-LL. This may be due to the fact that LBP can be triggered by a complex, multifactorial, and heterogeneous condition [40]. Clinically, we thought the test should be repeated or combined with other tests to compensate for the low sensitivity. However, this is the first paper we have, as far as we know, showing the cutoff points for spinopelvic alignment in LBP in SLSS. We believe the cut-off points of 47.0 mm for SVA and 30.5° for PI-LL may serve as indicators for diagnosing LBP in SLSS.

The major limitation of this study is that it is a cross-sectional study with a relatively small sample size. Further work is needed to confirm, longitudinally, that SVA and PI-LL are associated with LBP in LSS. However, the cut-off values of 47.0 mm for SVA and 30.5° for PI-LL determined through ROC analysis would be positive predictive values for LBP in SLSS. Past reports showed spinal decompression reduces leg pain, which eventually improved posture, and LBP [36, 38]. Conversely, we thought it was possible that LBP in cases with LSS symptoms but preserved spinopelvic alignment may not improve with surgery or rehabilitation, and the cut-off values in this study can be useful as criteria for determining this. Second, LBP in local spinal elements such as the intervertebral discs may not be completely ruled out. T2 values of the posterior annulus fibrosus were significantly lower in the LBP group on univariate analysis, although they were not significantly higher on multivariate analysis in this study. One possible mechanism of discogenic LBP is growth of the afferent fibres that surround the posterior annulus fibrosus into the disc [41]. We previously reported that the T2 value of the posterior annulus fibrosus at the L4–L5 level was lower in the CLBP group than in the control group [18]. Furthermore, we found that VAS scores were significantly negatively correlated with the T2 value of the posterior annulus fibrosus [18]. Future studies may need to include discography in cases where T2 values are low and painful discs are suspected. Thirdly, ORs were statistically significant, but close to 1, especially in the case of PI-LL. In this study, OR of PI-LL was calculated as 1.065, which means the person is 6.5% more likely to have LBP for every 1° increase in PI-LL. It is necessary that careful judgment should be applied in clinical applications. Finally, we did not analyse variables such as the duration of the disease and the impact of psychosocial factors.

## Conclusions

We compared lower extremity symptoms, bone mineral density, spondylolisthesis, range of motion, spinopelvic alignment, cross-sectional area and fat infiltration of the multifidus muscle, intervertebral disc degeneration, Modic changes, and facet joint degeneration between patients with high and low LBP who had LSS. The mean SVA was 56.1 mm and 29.8 mm (*p* < 0.01) and the mean PI-LL was 15.7° and 9.4° (*p* < 0.01) in the high and low groups, respectively. Multivariate logistic regression analysis revealed that (SVA; + 1 cm; OR, 1.331; 95% CI, 1.051 − 1.660) and pelvic incidence-lumbar lordosis (PI-LL; + 1°; OR, 1.065; 95% CI, 1.019–1.168) were significantly associated with LBP. ROC analysis revealed cut-off values of 47.0 mm of SVA and 30.5° of PI-LL, respectively. Our results indicated that SVA and PI-LL were significant predictors for LBP in SLSS. It is suggested that these parameters should be taken into consideration when assessing LBP in patients with SLSS. Further work is needed to confirm our findings and to see if we can use these factors to guide interventions.

## Data Availability

The datasets generated and/or analysed during the current study are not publicly available due to limitations of ethical approval involving the patient data and anonymity but are available from the corresponding author on reasonable request.

## Abbreviations

SLSS: Symptomatic lumbar spinal stenosis
LSS: Lumbar spinal stenosis
LBP: Low back pain
CLBP: Chronic low back pain
VAS: Visual analogue scale
LL: Lumbar lordosis
SVA: Sagittal vertical axis
PI: Pelvic incidence
MRI: Magnetic resonance imaging
OR: Odds ratio
CI: Confidence interval
ROC: Receiver operating characteristic
AUC: Area under the curve
